# Sialic Acids as Receptors for Pathogens

**DOI:** 10.3390/biom11060831

**Published:** 2021-06-02

**Authors:** Patrycja Burzyńska, Łukasz F. Sobala, Krzysztof Mikołajczyk, Marlena Jodłowska, Ewa Jaśkiewicz

**Affiliations:** Laboratory of Glycobiology, Hirszfeld Institute of Immunology and Experimental Therapy, Polish Academy of Sciences, R. Weigla 12, 53-114 Wroclaw, Poland; patrycja.burzynska@hirszfeld.pl (P.B.); lukasz.sobala@hirszfeld.pl (Ł.F.S.); krzysztof.mikolajczyk@hirszfeld.pl (K.M.); marlena.jodlowska@hirszfeld.pl (M.J.)

**Keywords:** sialic acids, receptors for pathogens, *CMAH* gene, phylogenetic tree

## Abstract

Carbohydrates have long been known to mediate intracellular interactions, whether within one organism or between different organisms. Sialic acids (Sias) are carbohydrates that usually occupy the terminal positions in longer carbohydrate chains, which makes them common recognition targets mediating these interactions. In this review, we summarize the knowledge about animal disease-causing agents such as viruses, bacteria and protozoa (including the malaria parasite *Plasmodium falciparum*) in which Sias play a role in infection biology. While Sias may promote binding of, e.g., influenza viruses and SV40, they act as decoys for betacoronaviruses. The presence of two common forms of Sias, Neu5Ac and Neu5Gc, is species-specific, and in humans, the enzyme converting Neu5Ac to Neu5Gc (CMAH, CMP-Neu5Ac hydroxylase) is lost, most likely due to adaptation to pathogen regimes; we discuss the research about the influence of malaria on this trait. In addition, we present data suggesting the *CMAH* gene was probably present in the ancestor of animals, shedding light on its glycobiology. We predict that a better understanding of the role of Sias in disease vectors would lead to more effective clinical interventions.

## 1. Introduction

Sialic acids (Sias) are the terminal carbohydrate moieties that occur on cell glycocalyx. They have been found in almost all forms of life, including humans and other mammals, but also in fungi, algae, bacteria and viruses [1,2], creating a host-specific “glycocalyx and sialome” on the cell surface. Most Sias are linked to glycoproteins, but in human neuronal cells, sialic acids are linked to sphingolipids (gangliosides) [3]. Sias mediate a wide variety of physiological and pathological processes, serving as ligands for cell adhesion molecules [4,5]. In fact, Sias are considered “self-associated molecular patterns” (SAMPS) cooperating with “self-pattern recognition receptors” (SPRR) [6]. Due to their negative charge, hydrophobicity and terminal position in sugar chains, they play an important role in cell stability and direct interactions with pathogens and toxins [4,5].

### 1.1. Structure of Sialic Acids

**Figure 1:**
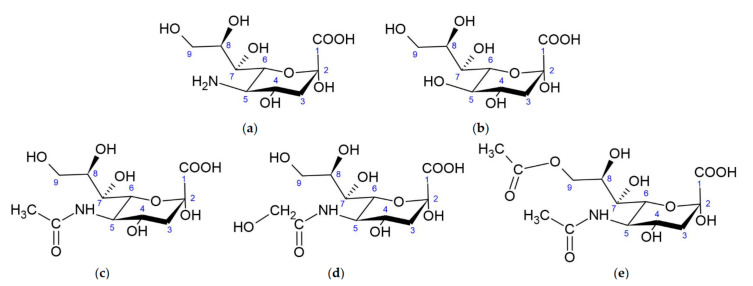
Structure of sialic acids: (**a**) Neuraminic acid, (**b**) 2-keto-deoxynonulosonic acid, (**c**) *N*-acetylneuraminic acid, (**d**) *N*-glycolylneuraminic acid and (**e**) *N*-acetyl-9-*O*-acetylneuraminic acid.

Sias are sugar [7] derivatives of neuraminic acid (nonulosonic acid [3]) (Figure 1a) usually present in glycoconjugates [8]. Neuraminic acid is chemically named 5-amino-3,5-dideoxy-d-glycero-d-galacto-non-2-ulopyranosonic acid (according to IUPAC 2020) and belongs to the 9-carbon α-keto carboxylic acids [3]. Three functional groups can be distinguished in Sias: a carboxyl group linked with the anomeric C-2 carbon (which making them ketoses), a three-carbon glycerol chain and an amino group linked to carbon C-5.

**Figure 2:**
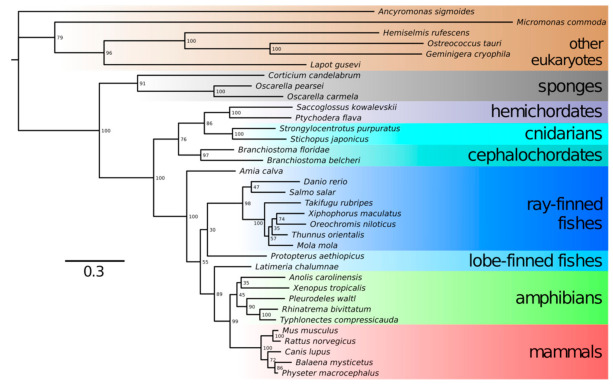
Single protein maximum likelihood tree of CMAH protein sequences from various species. Sponge CMAH proteins form a clade and are grouped together with other metazoan sequences. Sequences were aligned using MUSCLE [29], trimmed in Geneious 8.1.9 (BioMatters, Ltd., Auckland, NZ) [30] and the tree was made in IQTree v2.1.2 [31] with the substitution model (LG+I+G4) selected using ModelFinder [32]. Nonparametric bootstrap branch supports are shown next to the nodes. As evident from the non-basal position of cnidarians, single protein trees only approximate true phylogenetic relationships.

There are three major forms of neuraminic acid: 5-*N*-acetylneuraminic acid (Neu5Ac) (Figure 1c), 5-*N*-glycolylneuraminic acid (Neu5Gc) (Figure 1d) and 2-keto-deoxynonulosonic acid (Kdn) (Figure 1b), having a hydroxyl group at the C-5 position. In vertebrates, the formation of Sias is catalyzed by a bifunctional enzyme, glucosamine UDP-GlcNAc-2-epimerase/*N*-acetylmannosamine kinase (encoded by *GNE* gene), which converts UDP-GlcNAc (uridine diphosphate *N*-acetylglucosamine) to ManNAc-6-P (*N*-acetyl-mannosamine 6-phosphate) and UDP (uridine diphosphate) [9]. ManNAc-6-P is condensed with phosphoenolpyruvate (PEP), forming *N*-acetylneuraminate 9-phosphate (Neu5Ac-9-P), by Neu5Ac-9-P synthase. Then, Neu5Ac-9-P is dephosphorylated by a specific phosphatase, resulting in the release of Neu5Ac to the cytoplasm. In prokaryotes the condensation of ManNAc (*N*-acetylmannosamine) and PEP is catalyzed by an aldolase. The same pathway can use Man-6-P instead ManNAc-6-P to generate Kdn [3].

Sias can be modified by methylation, phosphorylation, sulfation and, most commonly, *O*-acetylation at C-4, C-7 or C-9. *O*-acetylated forms of sialic acids have been observed in bacteria, as well as eukaryotes (including humans) [10]. A neuraminic acid molecule may contain one to three *O*-acetyl residues, but *N*-acetyl-9-*O*-acetylneuraminic acid (Neu5,9Ac_2_) is the predominant form [11] (Figure 1). *N*-acetyl-4-*O*-acetylneuraminic acid (Neu5,4Ac_2_) was not found in humans, but it has gathered more interest recently as a receptor for pathogens infecting vertebrates [12,13]. More than 80 Sias have been reported in the literature [3,14].

The linkage configurations of Sias are α-2,3 or α-2,6 with galactose or *N*-acetylgalactosamine and α-2,8 or α-2,9 in polysialic acids. These bonds are created by specific sialyltransferases and polysialyltransferases, respectively [15]. The α-glycosidic bonds linking Sias to glycans are hydrolyzed by glycoside hydrolases named sialidases or neuraminidases (EC 3.2.1.18 [16]). In mammals, four sialidases (NEU1-NEU4) exist that differ in localization, specificity and function [17,18]. Many viruses produce sialidases, which increases their virulence or pathogenicity. Sialidases cleave Sias to enable the virus to attach to the host cell surface or to recognize the receptor by changing its structure or to uncover it. Transsialidases relocate Sias residues from host to pathogen cell surfaces to prevent its recognition by the host immune system. They may vary in substrate specificity, affecting the immune system or facilitating a release of progeny virions from cells [19,20,21]. Bacterial sialidases are treated as drug development targets [22,23].

### 1.2. Neu5Gc vs. Neu5Ac

The transformation of Neu5Ac into Neu5Gc occurs by hydroxylation of the methyl group in the acetyl moiety. This reaction is catalyzed by cytidine monophosphate-*N*-acetylneuraminic acid hydroxylase (CMAH) [24]. The *CMAH* gene was reported to be present in chordates, including lancelets, tunicates, reptiles and amphibians; most fish and a number of mammals but, also, in algae and bacteria [2]. Only a few deuterostome lineages do not have a functional *CMAH* gene: among mammals, the *CMAH* gene is nonfunctional in humans, New World monkeys, the European hedgehog, musteloids, several bats, the sperm whale, white-tailed deer and the platypus. Thus, these organisms cannot synthesize Neu5Gc, which may influence their susceptibility to parasites. According to previous analyses [2,25], *CMAH* was acquired by a deuterostome via lateral gene transfer from a microalga. Notwithstanding the question of deuterostome monophyly [26], we performed BLAST searches using an extensive database of eukaryotic protein sequences and recovered a full sequence of putatively functional CMAH from a sponge *Oscarella pearsei* and partial sequences from sponges *Oscarella carmela* and *Corticium candelabrum*. All of these sponges belong in the class Homoscleromorpha; no CMAH sequences were detected in any of the three other sponge classes. Phylogenetic trees prepared in IQTree 2 using representative animal and nonanimal CMAH protein sequences showed that sponge CMAH belonged within a sister group to other animal CMAH sequences, while nonanimal CMAH sequences were basal to them (Figure 2). The placement of sponge CMAH within this sister clade had full bootstrap support. It follows that the alga-to-deuterostome transfer hypothesis should probably be revised; the gene seems to have appeared in animals earlier. We did not detect CMAH in any choanoflagellates (the sister group to animals) or other nonanimal holozoan species but only in protists even less closely related to animals (Figure 2). If we assume sponges are the earliest known branch of animals [27], *CMAH* might have been one of the genetic novelties present in the lineage that led to animals, present in the last common ancestor of animals but not in the (earlier) last unicellular ancestor of animals [28]. We posit that Neu5Gc presence might have shaped the relationships between animals and their pathogens since the Metazoa kingdom evolved.

**Figure 3:**
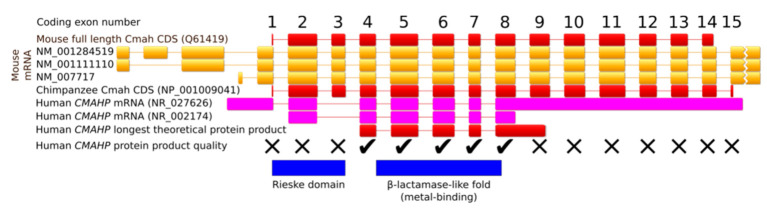
Organization of the *CMAH* gene. The human gene lacks coding exon 3 in comparison to the mouse and the chimpanzee genes. The ticks show exons that are putatively still functional, and the crosses denote exons that were damaged in the human pseudogene, resulting in either premature STOP codons or inactivating mutations in the protein product. The exons are drawn to scale relative to each other, and the introns were rescaled to the same length.

In comparison with other mammals, the human *CMAHP* pseudogene lacks exon 3 [33] (Figure 3). Two to three million years ago, a deletion of 92 bp changed the open reading frame (ORF) to cause a premature STOP codon, resulting in a 72-aa-long truncated protein [34,35]. The longest current human *CMAHP* ORF encodes a 247-aa protein containing only the C-terminal β-lactamase-like domain (Figure 3). Even if this protein was produced, the lack of the N-terminal Rieske domain erases CMAH activity. The full ancestral human CMAH protein was 590 aa long, identically to the chimpanzee enzyme. Inactivation of the *CMAH* gene in animals occurred many times in independent lineages. For example, in New World monkeys, separate events caused an inversion of exons 4-13, a deletion of exons 4-8 and a deletion of exons 10-13. The platypus harbors a premature stop codon in exon 5, and musteloids lost nine coding exons. Species with a nonfunctional *CMAH* gene only express Neu5Ac on the surface of their cells [2,36].

According to the catastrophic selection hypothesis, a factor related to CMAH enzyme loss might be a lethal virus recognizing Neu5Gc [37]. One pathogen that could play such a role might be the malaria parasite *Plasmodium*, for which Neu5Gc was an ancestral receptor [38]. Others speculated that anti-Neu5Gc antibodies made by *CMAH -*/*-* females could eliminate sperms from *CMAH*-positive males, leading to a depletion of functional CMAH in the population [39].

Although Neu5Gc is not produced by humans, its trace amounts can be detected on the surface of endothelial and epithelial cells, as well as in fetal tissues. The source of Neu5Gc is a diet rich in red meat and dairy products [40]. It is absorbed by micropinocytosis and subsequently incorporated into cells [41]. Moreover, Neu5Gc was also found in *Heamophilus influenzae*, a human commensal that can cause respiratory infections and meningitis. *H. influenzae* is also unable to synthesize Neu5Gc, but it can absorb the molecule from the diet of its host and incorporate this Sia into its surface oligosaccharides [42].

### 1.3. Interactions with Pathogens

Sialic acids can act as receptors for various pathogens underlying many infectious diseases (Table 1). For example, viruses can use Sias to attach to and enter cells, bacteria produce adhesins or toxins interacting with Sias on host cells and protozoa can use Sias to evade host immunity. In this review, we describe the known molecular interactions of Sias with various pathogens. For more detailed information, we refer to recent reviews in these topics [43,44,45,46,47,48,49].

## 2. Viruses

### 2.1. Influenza Virus

There are a several types of influenza viruses, all belonging to *Orthomyxoviridae* family. Influenza virus type A (IAV) causes seasonal disease in humans, but it was also responsible for the 1918 Spanish flu and 2009 swine flu pandemics [89]. In addition to humans, it can infect other animal species, including pigs, horses and birds. Influenza virus type B (IBV) causes almost the same disease symptoms as IAV, but although it is able to colonize animals (dogs or grey seals), it infects mainly humans and does not have pandemic potential [50]. Influenza virus type C (ICV) causes mild flu symptoms. The main reservoir of ICV are humans, but it is able to infect pigs as well. Influenza virus type D (IDV), whose reservoir is cattle, is harmless for humans [53].

The antigenic proteins that participate in infection of influenza viruses comprise of hemagglutinins (HA), neuraminidases (NA) [90] and the hemagglutinin esterase fusion protein (HEF) [54]. Sialic acids are receptors for all types of influenza viruses, which recognize them using HA. Both the type and the linkage of a sialic acid affect the infectivity of influenza viruses in a species-specific manner [51]. Avian IAV binds to α-2,3-linked Neu5Ac, which is present in the human lower respiratory tract, while human IAV binds mainly α-2,6-linked Sias, present in the upper respiratory tract. Thus, the location of infection depends on the binding preferences of HA. The binding depends on the length of the surface glycans, as well as their density of sialylation [91]. IBV infects mainly human upper respiratory tract, but some strains are able to infect the lower respiratory tract. Thus, it seems IBV also prefers α-2,6-linked Sias. NAs remove sialic acids, promoting viral attachment to cell surface and then its internalization by endocytosis [92]. It was found that some IAV strains can bind to Neu5Gc [93] on human epithelial cells, which may be incorporated from diet, but it does not work as a functional receptor. It was suggested that Neu5Gc may act as a decoy towards IAV: it is not susceptible to the viral sialidase, which makes endocytosis impossible. As a result, this binding suppresses infection in humans.

The *O*-acetyl form of Neu5Ac is the preferred receptor for ICV. The major player in ICV infection is hemagglutinin-esterase-fusion protein (HEF), which binds to Neu5,9Ac_2_ (α-2,3 or α-2,6 linkage) and removes the 9-*O*-acetyl group from the receptor using esterase activity. HEF catalyzes subsequent viral fusion with host cell membrane [46,53]. ICV and IDV HEF, used for infection, are structurally very similar [53], as well as the Infectious Salmon Anemia Virus (ISAV) HEF protein, which is specific to Neu5,4Ac_2_ and is not contagious for humans [12,13,94].

### 2.2. SV40

Simian virus 40 (SV40) belongs to polyomaviruses, and its primary reservoir are monkeys. People can be infected through aerosols or ingestion. SV40 was found to contaminate the polio vaccines, which were produced in 1950s and 1960s in monkey cells [95]. The virus has long been known to cause cancer in some species, including humans, most often mesothelioma and brain tumors. Cells in these tissues are nonpermissive to SV40 replication, but the virus can integrate into them and cause malignant transformation instead of cell lysis [96].

The natural SV40 receptor is GM1 ganglioside, a monosialotetrahexosyl glycosphingolipid. Upon binding of GM1 to the binding pockets formed by pentamers of viral capsid protein VP1, cholesterol-dependent endocytosis occurs [97]. It was found that SV40 binds to Neu5Gc-GM1 much tighter than to Neu5Ac-GM1 [98], which reduces its infectivity towards human cells [99].

### 2.3. Coronaviruses

Coronaviruses cause infections of respiratory and gastrointestinal tracts. They are classified into four genera: alpha-, beta-, gamma- and deltacoronaviruses [100]. All coronaviruses are able to infect mammals, but gamma- and deltacoronaviruses can also infect avian species. The coronaviruses most pathogenic for humans, the severe acute respiratory syndrome coronavirus (SARS-CoV) and Middle East respiratory syndrome coronavirus (MERS-CoV) are all betacoronaviruses. SARS-CoV-2, responsible for the 2019-21 pandemic [101], is more closely related to SARS-CoV than MERS-CoV and also belongs to the betacoronavirus genus. The main receptor for SARS-CoV viruses is angiotensin-converting enzyme 2 (ACE2), and for MERS-CoV, it is dipeptidyl peptidase 4 (DPP4 or CD26). In addition to DPP4, MERS-CoV recognizes sialic acids. It was shown that MERS-CoV Spike (S) protein preferentially binds to α-2,3-Neu5Ac, while its binding to α-2,6-Neu5Ac, Neu5,9Ac_2_ and Neu5Gc was low or nonexistent [56]. While sialic acid facilitates MERS-CoV infection, in the case of SARS-CoV and SARS-CoV-2, the presence of sialic acids seems to restrict the infection. It was shown that the NA treatment of lung epithelial cells significantly promoted the replication and infection of both viruses, wherein SARS-CoV-2 was affected to a lesser extent. These results suggested a precluding role of ACE2 receptor Sias for SARS-CoV -2 binding. The increased SARS-CoV-2 S protein binding to ACE2, treated by NA, indicates a different role of Sias on SARS-CoV-2 binding and infection [102]. Thus, viral attachment likely occurs in a two-step fashion; the first low affinity Sias binding is followed by the higher affinity protein receptor binding [103].

Other human betacoronaviruses, OC43 and HKU1, recognize 9-*O*-acetylated sialic acid [55,104]. These viruses produce the sialate-*O*-acetylesterase protein (HE), which contains an *O*-acetylated sialic acids-binding domain and sialate *O*-acetylesterase domain. It was suggested that the HE protein evolved from the ICV HEF protein, but its binding activity is lost. This enzyme derivatizes *O*-acetylated sialic acids at a later stage of infection to prevent irreversible binding of progeny virions to the 9-*O*-acetylated Sias decoys. Mouse Hepatitis Virus (MHV) is another betacoronavirus producing HE protein similar to ICV HEF, but like ISAV, it recognizes Neu5,4Ac_2_ and is not able to infect humans [12,13,105,106].

### 2.4. Human Parainfluenza Viruses

Human parainfluenza viruses (HPIVs) are common respiratory pathogens belonging to the *Paramyxoviridae* family. They are divided into four serotypes (HPIV-1–HPIV-4), the last of which has two major subtypes (HPIV-4A and HPIV-4B) [107]. HPIVs cause lower and upper respiratory tract infections, mainly in newborns and young children, but also in elderly and immunocompromised patients. The most common symptoms include cold-like symptoms, tracheobronchitis, bronchiolitis, laryngotracheobronchitis and pneumonia. HPIV infections are associated with a wide range of neurological and cardiovascular illnesses, i.e., febrile seizures, meningoencephalitis, myocarditis, pericarditis and bradycardia [108].

One of the six essential proteins encoded by HPIV RNA is a membrane-anchored hemagglutinin neuraminidase (HN), which uses sialic acid as a receptor in host cell entry. The HN glycoprotein is also involved in the budding and release of new virions from the infected cell by Sias residue cleavage [109]. The viral HN binds predominantly to oligosaccharides terminated with α-2,3-linked Neu5Ac, but it seems that HPIV-1 HN can cleave α-2,8-linked Sias from α-2,3-linked Sias without binding to it [57]. Moreover, HPIV-3 HN shows ability to bind to α-2,6-linked Neu5Ac and α-2,6-linked Neu5Gc [58,59].

## 3. Bacteria

### 3.1. Salmonella enterica

*Salmonella enterica* is a Gram-negative, rod-shaped bacterium. The species is divided into six subspecies and over 2600 serovars [110]. One of them, *Salmonella* Typhi, is a bacterium causing typhoid fever in humans with such symptoms as fever, headache, rash and abdominal pain. Its toxin belongs to the two-subunit AB_5_ six-component toxin type, unique seven-component structure (A_2_B_5_). The A subunits have enzymatic activity: deoxyribonuclease CdtB and ADP ribosyltransferase PltA, while the B subunits (PltB) bind to glycoprotein receptors with terminal Neu5Ac but not Neu5Gc [60]. The expression of the highly immunogenic *S.* Typhi toxin is substantially upregulated after bacterial entry into the host cells [111]. In species that express Neu5Gc, *S.* Typhi is unable to multiply. The only exception is the chimpanzee, in which *S.* Typhi was identified, but no disease symptoms have been observed.

Another example is *Salmonella* Typhimurium that causes bacteremias and acute gastroenteritis, with symptoms such as diarrhea [112,113]. Its toxin ArtAB, which is similar to *S.* Typhi toxin, is an important virulence factor causing symptoms, which are an effect of its immunogenicity that triggers the immune system response [114]. It was shown that Neu5Ac is a potential receptor for *S.* Typhimurium [61]. Moreover, *S.* Typhimurium LT2 sialidase prefers α-2,3-linked over α-2,6-linked sialic acids, and its ability to cleave Neu5Gc is low [115].

### 3.2. Vibrio cholerae

Cholera, caused by *Vibrio cholerae*, is a disease specific only to humans [116], whose symptoms include diarrhea, vomiting and muscle cramps. The bacterium has three cytotoxicity factors: TCP (toxin-coregulated pilus) responsible for the colonization of small intestine, CTx (cholera toxin) and VcN (*V. cholerae* neuraminidase) [62,117]. CTx belongs to the AB_5_ family and recognizes monosialylated Neu5Ac-GM1 or Neu5Gc-GM1. However, the majority of gangliosides on the surface of human epithelial cells are di- and trisialo-derivatives. *V. cholerae* uses a sialidase to hydrolyze α-2,3- and α-2,8-linked sialic acids, turning them into GM1. It was shown that Neu5Gc bound to GM1 is less susceptible to enzymatic hydrolysis in comparison to Neu5Ac. Thus, it seems that the species adapted to the human-specific loss of Neu5Gc, which underlies our susceptibility to this pathogen.

### 3.3. Pseudomonas aeruginosa

*Pseudomonas aeruginosa* is a Gram-negative, rod-shaped bacteria, often found in plants, soil and water environments and, sometimes, in human bodies. *P. aeruginosa* is an opportunistic pathogen with natural resistance to antibiotics [118,119], causing hospital-acquired infections, e.g., pneumonia, meningoencephalitis and sepsis [63]. It infects mostly the respiratory and urinary tracts, blood, burn wounds and the outer ear. Its ability to form biofilms, combined with quorum sensing mechanisms [120], is associated with extreme drug tolerance [121].

The main virulence factors are the single flagellum, pili and components of the outer plasma membrane that participate in adhesion to the host airway epithelia [122]. Moreover, *P. aeruginosa* produces toxins: pyocyanin [123], lipopolysaccharide and exotoxin A [124]. The flagellum interacts with Toll-like receptors TLR-5 and TLR-2 [125,126], mucins [127] and heparan sulfate proteoglycans [122,128], while the pili bind to N-glycans [129]. Furthermore, *P. aeruginosa* interacts with laminin [130,131] and integrins [132,133,134]. Both the pili and the flagellum recognize asialo-monogangliosides (GM1 or GM2), which expose a GalNAcβ1-4Gal moiety [64]. Such binding promotes an inflammatory response [126], which is especially threatening to individuals with cystic fibrosis because of an increase of asialo-GM1 expression on the surfaces of airway cells [135]. Another receptor of *P. aeruginosa* is the sialyl-Lewis x antigen with terminal α-2,3-Neu5Ac [65], and its importance in the context of cystic fibrosis is similar.

### 3.4. Helicobacter pylori

*Helicobacter pylori* is a helical-shaped Gram-negative bacterium able to survive in extreme acidic conditions; hence, its niche is usually the stomach lining. It is a common cause of ulcers and gastritis [136], which, untreated, can lead to gastric cancer [137]. *H. pylori* has flagella, which are necessary for bacterial motility in gastric mucus [138]. The bacterium produces Ni^2+^-dependent urease [139], which hydrolyzes urea into ammonia to neutralize the acidity and create an optimal environment to survive in the stomach. Other virulence factors of *H. pylori* are adhesins, some of which recognize sialic acids. Adhesin SabA prefers *N*-acetyllactosamine-based gangliosides with terminal α-2,3-linked Neu5Ac (including the sLe^x^antigen) over α-2,6-linked Neu5Ac and terminal Neu5Gc [66,67,68,69]. Protein HpaA is an adhesin potentially capable of binding sialic acids [70,71], but its specificity is not known yet.

### 3.5. Haemophilus influenzae

*Haemophilus influenzae* is an opportunistic Gram-negative coccobacillus bacterium. Depending on its ability to form polysaccharide capsules, its strains may be divided to typeable (encapsulated) and non-typeable (unencapsulated) [140]. The encapsulated strains are categorized into six serotypes (from a to f), of which serotype b is considered the most virulent [141]. *H. influenzae* mainly causes respiratory tract infections, especially in children and the elderly, chronic obstructive pulmonary disease in cystic fibrosis patients [142,143], sinusitis [144] and pneumonia. It can also cause conjunctivitis [145] and otitis media (middle ear inflammation) [146].

*H. influenzae* strains have various factors participating in infection, playing a role in interactions with host cells [147,148,149,150,151]. They include adhesins [152,153] and other components anchored to its outer membrane: pili [154,155], IgA proteases [156] and lipooligosaccharide (LOS) [157]. It was shown that *H. influenzae* contains α-2,6-Neu5Ac [73], but the bacterium is not able to synthesize Sias and, instead, uses those absorbed from its environment [74]. Dietary Neu5Gc can be also incorporated into the LOS, but Neu5Ac is up to four times more favored [72]. It was suggested that *H. influenzae* mimics the sialylation pattern of host mucin [73].

### 3.6. Clostridium botulinum

*Clostridium botulinum* is a rod-shaped, Gram-positive bacterium with an ability to sporulate [158]. It is an obligate anaerobe thar lives in soil, dust and sediments, but it is also able to infect animals (including humans) through food. Botulism is a disease caused by botulinum neurotoxins. Its main symptom is a descending flaccid paralysis, which, if left untreated, can result in death.

Botulinum neurotoxins are metalloproteases that specifically cleave SNARE (soluble *N*-ethylmaleimide-sensitive factor attachment protein receptor) proteins in neurons, which results in the inhibition of neurotransmission [159]. Based on its antigenic properties and amino acid sequence, the botulinum toxins are classified into seven subtypes (A–G). Subtypes A, B, E and (rarely) F cause botulism in humans [160], while subtypes C and G affect birds and other animals. It was shown that types A-D and G toxins associate in complexes with hemagglutinins (HA) [75,76,77], which enables them to bind to gangliosides with terminal sialic acid. In some cases, such binding is critical for infectivity [78,161]. It is known that complexes of botulinum toxin and HA recognize Neu5Ac, but whether this happens with Neu5Gc as well is not known.

### 3.7. Streptoccocus pneumoniae

*Streptococcus pneumoniae*, also known as pneumococcus, is a commensal, Gram-positive, spherical bacterium. It may, however, cause pneumonia and other infections such as otitis media, sinusitis, meningitis and bronchitis [162,163], especially in children, the elderly and in patients with immunodeficiency. *S. pneumoaniae* produces various virulence factors, including surface enzymes, polysaccharide capsules, pili, choline-binding proteins, lipoproteins and toxin pneumolysin [164], as well as sialidases. These glycoside hydrolases can cleave the T-antigen, a mucin disaccharide normally covered by a terminal sialic acid, and are specific to both α-2,3- and α-2,6-linked Neu5Ac. The T-antigen is processed to galactose and *N*-acetylgalactosamine [80], which elicits an IgM response, leading to pneumococcal hemolytic uremic syndrome (HUS). Additionally, it was shown that Neu5Ac is preferred over Neu5Gc [79].

### 3.8. Escherichia coli

*Escherichia coli* is a species of Gram-negative, rod-shaped, commensal bacteria naturally occurring in the colonic microbiota of humans and animals, transmitted by water and sediments [165]. Its vast genetic diversity causes differences in its pathogenicity [166]. The disease-causing strains mainly infect the gastrointestinal and the urinary tracts. The major virulence factors of *E. coli* are pili [167], K- and O-antigens [168], adhesins [169], lipopolysaccharide [170], hemolysin [171] and toxins. The subtilase cytotoxin (SubAB) of Shiga toxin-producing *E. coli* (STEC) can damage the internal organs, and the infection caused by it is characterized by acute kidney failure, thrombocytopenia and microangiopathic hemolytic anemia [82]. SubAB belongs to AB_5_ toxins and binds to receptors by pentameric B subunits. The receptors are sialylated glycolipids with terminal α-2,3-Neu5Gc, to which the affinity is 20 times higher than to α-2,3-Neu5Ac and 30 times higher than to α-2,6-Neu5Gc [81].

## 4. Protozoa

### 4.1. Trypanosoma cruzi

*Trypanosoma cruzi* is a parasite causing Chagas disease, affecting mainly Latin America but spreading worldwide due to human migration and, also, the specific way of pathogen transmission. The vectors of *T. cruzi* are certain species of three genera of blood-sucking triatomine insects: *Triatoma*, *Panstrongylus* and *Rhadonius,* also called “kissing bugs”. Other possible routes of infection are: mother-to-child transmission, blood and blood products or contaminated food and drink. The major disease symptoms involve cardiac and digestive manifestations [172]. A protein important in *T. cruzi* infection is gp85—a transsialidase (TcTS) [173] that hydrolyses α-linked Sias present on the host cell surface and transfers them to mucin molecules on the parasite cell surface. Such a mechanism prevents pathogen recognition by the host immune system [174]. TcTS is specific to α-2,3-linked Sias on the donor cells, and it recreates the same linkage on the pathogen cells, where the acceptor is a terminal β-galactopyranosyl group. TcTS is able to transfer both Neu5Ac and Neu5Gc [175].

### 4.2. Entamoeba histolytica

*Entamoeba histolytica* is an anaerobic amoebozoan [176] belonging to the Entamoebidae family (Archamoebae). As a human pathogen, it causes amoebiasis with symptoms related to the gastrointestinal tract, with possible complications that include liver, lung or brain abscesses [177]. Foodborne infection occurs by ingestion food or water contaminated with *E. histolytica* cysts. Their cell wall structures allow them to survive in the acidic environment of the host stomach. In the small intestine, the cysts release motile trophozoites that colonize the colon and then differentiate into cysts that are excreted in the feces [178,179]. It was shown that trophozoites bind to host mucins in a sialic acid-dependent manner [87]. A transmembrane sialidase on the *E. histolytica* cell surface [180] uncovers the receptor before it can be recognized by the *E. histolytica* toxin. The sialidase substrates are α-2,3-Neu5Ac and colonic acid (a homopolymer of α-2,8-Neu5Ac), but α-2,6-Neu5Ac is not hydrolyzed [181].

### 4.3. Toxoplasma gondii

*Toxoplasma gondii* is a parasitic protozoan from phylum Apicomplexa that is able to infect humans and other warm-blooded animals. It is dangerous especially for immunocompromised individuals and developing fetuses. *T. gondii* encysts and establishes chronic infections in the brain, the heart and skeletal muscles [182,183]. While its life cycle contains both sexual and asexual phases, the only host where it can reproduce sexually is the cat. Inside the feline gastrointestinal tract, *T. gondii* differentiates into gametocytes that are then excreted in the feces. One of the major virulence factor of *T. gondii* are proteins produced by micronemes (apicomplexan parasites secretory organelles), and they recognize sugar chains on the host cell surface; thus, Sias are important molecules involved in infections. Toxoplasma microneme protein 1 (TgMIC1) has a high affinity for terminal α-2,3-linked Sias and a low affinity for α-2,6-linked Sias [184]. Another protein, Toxoplasma microneme protein 13 (TgMIC13), shows an affinity for 4-*O*-acetylated Sias and the less well-known α-2,9-linked Sias [185]. Recently, it was shown that TgTCP-1 chaperonin [186] and tachyzoite surface Sia-binding protein 1 (TgSABP1) interact with Sias on the host cell surface, and sialic acid ablation by sialidase affected the interaction [187].

### 4.4. Plasmodium

The malaria parasites from the genus *Plasmodium* also take advantage of the ubiquity of Sias on the surface on red blood cells to invade their hosts. *Plasmodium falciparum* is responsible for infecting humans, while African apes harbor at least six species of *Plasmodium*, three of which primarily infect chimpanzees (*P. reichenowi, P. gaboni* and *P. billcollinsi*), while the other three infect gorillas (*P. praefalciparum, P. blacklocki* and *P. adleri*) [188,189].

**Figure 4:**
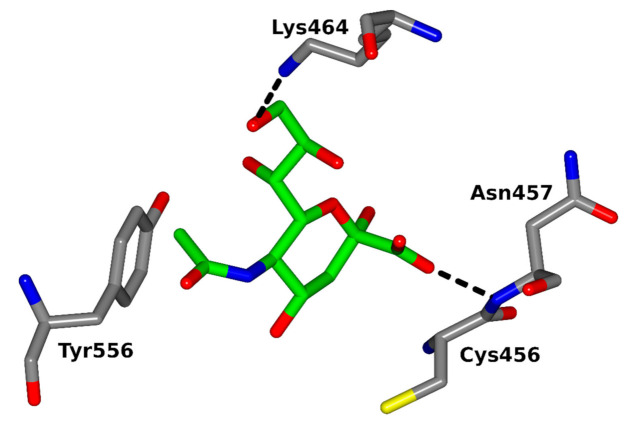
The F2-binding pocket of the PfEBA-140 ligand with the bound *N*-acetylneuraminic acid. The amino acid residues and backbone atoms involved in sugar binding are shown (according to [205], modified). Figure made in ccp4mg [208] using PDB ID 4JNO.

Several proteins expressed by *Plasmodium* merozoites are involved in the erythrocyte invasion, including two multigene families: erythrocyte-binding ligands (EBL) and reticulocyte-binding ligands (RBL) [190,191]. Four functional *P. falciparum* EBL proteins were identified so far [192]: erythrocyte-binding antigen-175 (EBA-175), erythrocyte-binding antigen-181 (EBA-181), erythrocyte-binding ligand-1 (EBL-1) and erythrocyte-binding antigen-140 (EBA-140). The well-studied *P. falciparum* EBA-175 merozoite ligand [83] recognizes α-2,3-linked Sias presented on clusters of O-linked glycans attached to erythrocyte surface glycophorin A (GPA) [193,194]. The EBA-140 homologous merozoite ligand [195,196,197] was shown to bind glycophorin C (GPC) [84,198,199,200], which is a minor erythrocyte sialoglycoprotein [201]. It was proposed that the receptor for EBA-140 ligand is a cluster of N- and O-linked sialylated glycans on the GPC molecule [202,203] (Figure 4). Thus, Sias seem to be necessary for the recognition of both EBA receptors [204,205,206,207].

Homologs of the PfEBL merozoite ligands were identified in ape parasites, including *P. reichenowi* [208]. Expression of *P. falciparum* and *P. reichenowi* EBA-175 and EBA-140 on the surface of African green monkey (COS7) cells showed some Sias host-specific preferences for erythrocyte binding [209]. The preferred ligand for *P. falciparum* EBA-175 and EBA-140 was Neu5Ac, while *P. reichenowi* proteins bound preferentially to Neu5Gc. Thus, it has been argued that the expression of different Sias is a primary factor responsible for the species-specific binding of human and ape parasites [39,209].

Further studies on the *P. falciparum* EBA-175 ligand specificity confirmed that it associates with GPA only when Neu5Ac linked to α-2,3-Gal is present [210]. However, the strong binding of EBA-175 to Neu5Gc monosaccharide was also observed.

In another approach, human ex vivo cultured erythrocytes (cRBCs) were modified by introducing Neu5Gc on the cell surface through the expression of chimpanzee *CMAH* [211]. The presence of Neu5Gc on human cRBCs significantly increased *P. knowlesi* merozoite invasion. However, in contrast to *P. knowlesi*, which is a parasite of macaques, the human parasite *P. falciparum* invaded normal and modified cRBCs at a similar level, which suggested that *P. falciparum* can utilize both Neu5Ac and Neu5Gc. This result supported the bespoke hypothesis that PfEBA-175, the major sialic acid-dependent invasion ligand, does not discriminate between Neu5Ac and Neu5Gc.

EBA-165, another homologous merozoite ligand dependent on Sias binding, is present in all ape-infective *Plasmodium* species, but due to a mutation causing a frameshift, it is not present in *P. falciparum* [212]. Consistent with the previous findings [211], it was confirmed that the PfEBA-175 ligand was able to bind to glycans containing Neu5Ac and Neu5Gc. By contrast, *P. reichenowi* EBA-165 and the full-length, corrected *P. falciparum* EBA-165 (cP.f. EBA-165) bound only to glycans containing Neu5Gc.

In summary, there is clear preference for Neu5Gc in all studied *Plasmodium* ape species (*P. reichenowi* and *P. knowlesi*), while the human parasite *P. falciparum* shows a specificity towards both Neu5Gc and Neu5Ac. Consequently, the gain/loss of function mechanism may serve as a host switch for *P. falciparum*, because *P. falciparum* has gained the ability to bind Neu5Ac without losing the binding specificity for Neu5Ac. It might have implications for the emergence of *P. falciparum* as a major deadly human pathogen. While the EBA Sias preference hypothesis is intriguing, it may not represent the complete story of *Plasmodium* merozoites specificity.

## 5. Conclusions

The glycocalyx of human cells differs from many other mammals by the lack of the Neu5Gc and the presence of its precursor Neu5Ac. Humans are not alone in this loss. Several other species of mammals have independently developed different loss-of-function mutation in the *CMAH* gene. This convergent evolution of the vertebrate-specific Neu5Gc-containing glycans represents an evolutionary change in the use of Sias as receptors for pathogens. A consequence of this change was the change in pathogen regimes; thus, pathogens that can bind Neu5Ac would have a preference for humans. Indeed, a number of human-specific pathogens evolved a specificity for Neu5Ac. These include most influenza viruses, bacteria and the human malaria parasite *P. falciparum*. Presumably, pathogens that require Neu5Ac could differentially expand in humans. Thus, a better understanding of human infectious disease mechanisms based on Sias would allow for new drug designs; for example, the Sias structural analogs currently used as specific inhibitors in influenza virus infection treatment.

## Figures and Tables

**Table 1:** Pathogens exploiting sialic acids as receptors: viruses, bacteria and protozoa.

Pathogen/Toxin	Disease	Ligand on Pathogen	Type of Sialic AcidBound/Incorporated	Symptoms in Humans	References
Avian influenza virus A	Flu	HA	α-2,3-Neu5Ac	Mild symptoms, fever, cough, sore throat, muscle anches	Van de Sandt et al., 2015 [50]
Human influenza virus A and B	Flu	HA	α-2,6-Neu5Ac/Neu5Gc ^1^	Fever, cough, runny or stuffy nose, tiredness, sore throat, muscle anches	de Graaf and Fouchier 2014 [51]
Human influenza virus C	Flu	HEF	O-acetyl form of α-2,3- or α-2,6-Neu5Ac	Mild cough, fever, malaise	Wang and Veit 2016 [52]
Human influenza virus D	Flu in animals	HEF	O-acetyl form of α-2,3- or α-2,6-Neu5Ac	No symptoms detected in humans	Su et al., 2017 [53]
SV40 virus	Carcinogenic in humans	VP1	NeuAc (weak binding in humans) and NeuGc (strong binding in simians) on GM1 glycolipid	Mesothelioma, osteosarcoma, pediatric and adult brain tumors and non-Hodgkin lymphomas	Shah 2004 [54]
Human *betacoronaviruses* - OC43 and HKU1 - MERS	Colds, human pulmonary infectious, lethal encephalitis (only OC43) Middle East respiratory syndrome	Spike protein	9-O-acetylated Neu5Ac α-2,3-Neu5Ac	Fever, weakness, abdominal pain, rhinitis, and sore throat, sometimes severe pneumonia (HKU1), vomiting, diarrhea, abdominal pain (OC43) Fever, cough, breath shortness, pneumonia, sometimes diarrhea	Hulswit et al., 2018 [55] Li et al., 2017 [56]
Human parainfluenza viruses	Respiratory tract infections	HN	α-2,3-Neu5Ac α-2,6-Sias (HPIV-3)	cold-like, bronchiolitis, tracheobronchitis, laryngotracheobronchitis, pneumonia	Amonsen et al., 2007 [57]; Suzuki et al., 2001 [58]; Zhang et al., 2005 [59]
*Salmonella enterica* - *S.* Typhi - *S*. Typhimurium (LT2)	Typhoid fever Bacteremia and acute gastroenteritis	B subunit of toxin	α-2,3-Neu5Ac α-2,3-Neu5Ac > α-2,6-Neu5Ac and low ability to Neu5Gc	Fever, headache, dry cough, loss of appetite fever, nausea, vomiting, diarrhea diseases	Gao et al., 2017 [60] Kappala et al., 2018 [61]
*Vibrio cholerae*	Cholera	B subunit	Neu5Ac and Neu5Gc on GM1	Watery diarrhea, vomiting, rapid heart rate, low blood pressure	Muanprasat and Chatsudthipong 2013 [62]
*Pseudomonas aeruginosa*	Pneumonia, meningoencephalitis and sepsis	Pili and flagellum Flagellum	asialo-GM1 and asialo-GM2 with GalNAcβ1-4Gal moiety sialyl-Le^x^ epitope with terminal α-2,3-Neu5Ac	Pneumonia and urinary tracts-related symptoms, such as cough, fever, difficulty breathing, swelling	Bassetti et al., 2018 [63]; Krivan et al., 1988 [64]; Xia et al., 2006 [65]
*Helicobacter pylori*	Ulcer, gastritis and gastric cancer	SabA adhesion HpaA	*N*-acetyllactosamine-based gangliosides with terminal α-2,3-linked Neu5Ac > Neu5Gc and α-2,6-linked Neu5Ac Sialic acid	Ache or burning pain in abdomen, nausea, loss of appetite, bloating	Evans et al., 1988 [66]; Mahdavi 2002 [67]; Roche et al., 2004 [68]; Benktander et al., 2018 [69]; Bennett and Roberts 2005 [70]; Carlsohn et al., 2006 [71]
*Haemophilus influenzae*	Respiratory tract infections: pneumonia, sinusitis; conjunctivitis and otitis media	Lipooligosaccharide (LOS)	Incorporation of α-2,6-Neu5Ac (from human host) and/or dietary Neu5Gc into LOS	Fever, chills, diarrhea, anxiety, difficulty breathing, cough, muscle pain	Ng et al., 2019 [72]; Greiner et al., 2004 [73]; Johnston et al., 2008 [74]
*Clostridium botulinum*	Botulism	Botulin/HA complex	Gangliosides, prefer α-2,3-Neu5Ac > α-2,6-Neu5Ac	Double and blurred vision, dropping eyelids, difficulty swallowing, dry mouth, a thick-feeling tongue	Fujinaga et al., 2000 [75]; Rummel et al., 2007 [76]; Sagane et al., 2016 [77]; Sugawara et al., 2015 [78]
*Streptococcus pneumoniae*	pneumonia, otitis media, sinusitis, meningitis and bronchitis	Sialidases: NanA-C	Cleavage of α-2,3- and α-2,6-Neu5Ac	Fever, cough, shortness of breath, increased sensitivity to light, ear pain, hearing loss	Hatcher et al., 2016 [79]; Coats et al., 2011 [80]
SubAB toxin produced by Shiga toxin-produced *Escherichia coli*	Hemolytic-uremic syndrome (HUS)	B subunit	α-2,3-Neu5Gc > α-2,3-Neu5Ac > α-2,6-Neu5Gc	Extreme fatigue, decreased urination or/and blood in the urine, swelling of the legs, feet, ankles, high blood pressure	Byres et al., 2008 [81]; Seyahian et al., 2017 [82]
*Plasmodium falciparum*	Malaria	EBA-175, EBA-140	α-2,3-Neu5Ac on GPA and GPC, respectively	Fever, headache, vomiting, tiredness	Tolia et al., 2005 [83]; Rydzak et al., 2015 [84]
*Plasmodium reichenowi*	Malaria in chimpanzee	EBA-140	Neu5Gc on chimpanzee GPD	Malaria-like symptoms	Zerka et al., 2017 [85]
*Trypanosoma cruzi*	Chagas disease	No ligand, *trans*-sialidase	Trans-sialidase transfers α-2,3-linked Sias from host to surface mucin-like glycoproteins	Fever, malaise, headache, enlargement of the liver, spleen, lymph nodes	Freire-de-Lima et al., 2012 [86]
*Entamoeba histolytica*	Amoebiasis	Sialoglycoproteins	α-2,3-Neu5Ac	Stomach pain and cramping, loose feces, in severe form bloody feces and fever	Kato et al., 2013 [87]
*Toxoplasma gondii*	Toxoplasmosis	TgMIC1 TgMIC13	α-2,3-linked Sias 4-*O*-acetylated Sias and α-2,9-linked Sias	Influenza-like symptoms with swollen lymph glands or muscle aches, damage of brain or eye, reduced vision	Nishikawa et al., 2013 [88]

^1^ Human IAV may binds to dietary Neu5Gc.

## Data Availability

The data used to generate the phylogenetic trees, as well as all the CMAH protein sequences considered in this review, are available on Zenodo under DOI: https://doi.org/10.5281/zenodo.4585495.
